# Evaluation of Renal Function in Pregnant Women with Malaria: A Case-Control Study in a Mesoendemic Area

**DOI:** 10.1155/2017/6030943

**Published:** 2017-03-07

**Authors:** Justice Afrifa, Samuel Essien-Baidoo, Albert Baffour Gyau, Richard Kobina Dadzie Ephraim

**Affiliations:** Department of Medical Laboratory Technology, School of Allied Health Sciences, College of Health and Allied Sciences, University of Cape Coast, Cape Coast, Ghana

## Abstract

*Background.* Malaria is known to have devastating effects on mortality in tropical and subtropical regions with the effect being magnified in people with weakened immunity such as those in pregnancy. We assessed the effect of malaria on renal function of pregnant women receiving antenatal care in a mesoendemic area of Ghana.* Methodology.* A case-control study that enrolled a total of 100 pregnant women (50 with confirmed gestational malaria as cases and 50 without malaria as controls). Sociodemographic characteristics, obstetric history (obtained with a questionnaire), urea, creatinine, sodium, and potassium were analyzed using a chemistry automated analyzer.* Results.* Plasma urea and creatinine were significantly increased (*P* = 0.0003 and *P* < 0.0001, resp.) among cases compared to the controls. Also the levels of urea (*P* = 0.033), creatinine (*P* = 0.032), and parasitaemia (0.016) were significantly increased with increasing gestational age.* Conclusion.* Malaria has a significant impact on renal function (most importantly, urea and creatinine) and is also significantly associated with increasing gestational age among our study participants.

## 1. Introduction

Malaria during pregnancy is a major public health problem in tropical and subtropical regions with 30.3 million African women becoming pregnant in malaria endemic areas, and, in spite of this, only a fraction of these women have access to effective interventions [1, 2]. It is also known that pregnant women are more susceptible to malaria than their nonpregnant counterparts [3]. This is because pregnant women with malaria have a reduced immunity due to their state [4].

Pregnancy and malaria mutually tend to present aggravating conditions. The physiological and the pathological changes due to malaria have a synergistic effect on the patient which makes life difficult for the mother, child, and the treating physician [5]. Malaria during pregnancy has been known to have several effects on pregnancy outcome, and this includes increased neonatal mortality by lowering birth weight as well as the induction of premature births [6].

One of the dreaded causes of foetal mortality and morbidity is malaria in pregnancy. Anaemia, hypoglycemia, and other complications associated with malaria can adversely affect the foetus. Interestingly, malaria caused by falciparum has been shown to cause problems leading to foetal mortality up to 15% [7, 8]. Spontaneous abortion, placental insufficiency, and still births have also been observed as a consequence of malaria in pregnancy [9].

A contemporary study to understand the relationship between malaria and renal failure found a significant increase in serum creatinine with 67.14% of the patients recording serum creatinine level above normal. The researchers also reported that 52.86% of their participants had their serum urea level above the normal range which the World Health Organization describes as an indication of renal failure [10].

Even where antenatal screening for malaria is a national policy, the WHO criteria acknowledge that some biochemical and haematological features should raise suspicion of severe malaria and renal screening for malaria in pregnant women should sporadically be implemented at best [11].

Few estimates exist as to how well this problem is being addressed in areas of the world where screening and treatment could have an enormous impact [12]. Meanwhile, in spite of the linkage of malaria to the renal integrity in malaria infection, there is limited information about the effect of these biochemical renal parameters among pregnant women in Ghana especially in mesoendemic areas. Besides, the cross-sectional nature of available studies limits the scope with which various results could be interpreted. However, the fraction of adverse effects of malaria infection that can potentially be averted in pregnancy is expected to be high especially in relation to renal biochemistry. We therefore sought to assess the effect of malaria on renal function of pregnant women receiving antenatal care in a mesoendemic area of Ghana.

## 2. Methods

### 2.1. Study Design/Study Site

**Figure 1:**
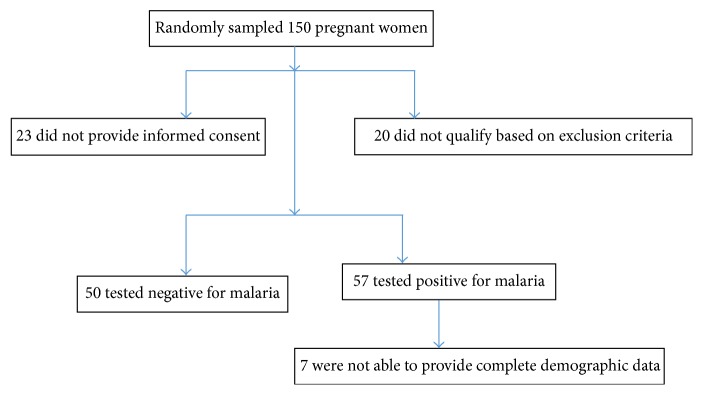
Flow chart of sample selection and inclusiveness.

This hospital-based case-control study was carried out between October, 2015, and April, 2016, at the Koforidua Polyclinic. A total of 100 patients (50 with gestational malaria as cases and 50 healthy pregnant women as controls) were recruited for this study. The flow chart for sample selection and inclusiveness is shown by Figure 1.

### 2.2. Inclusion Criteria

Pregnant women with singleton pregnancies receiving antenatal care at the study center were eligible to participate in this study.

### 2.3. Exclusion Criteria

Exclusion criteria were participants with preexisting renal diseases, chronic kidney disease, hypertension and diabetes mellitus, human immunodeficiency virus, and acquired immune deficiency.

### 2.4. Ethical Considerations

Ethical clearance for the study was obtained from the University of Cape Coast Institutional Review Board (UCC/IRB) and from the authorities of Koforidua Polyclinic. Consent was sought from participants having explained to them the purpose of the research and its relevance. Participants were made to willingly opt out anytime they felt uncomfortable or had a change of mind.

### 2.5. Collection of Obstetric Data

With the aid of a questionnaire a resident or an intern nurse obtained sociodemographic characteristics and obstetric history (parity, gravidity) of consented participants.

### 2.6. Sample Collection and Processing

Five (5) milliliters of venous blood was taken from each patient for the blood film and the biochemistry assay. Blood smears were prepared and stained for the examination of malarial parasites using appropriate techniques [14]. The thick and thin films were analyzed for the number of parasites per 200 white blood cells [15]. The level of parasitaemia was graded as low (<1000 parasites/*μ*L of blood), moderate (1000–9999 parasites/*μ*L of blood), and severe (≥10,000 parasites/*μ*L of blood) [16]. Biochemical analysis was performed with the BT-3000 Analyzer. The reaction principles for the estimation of urea and creatinine were based on the urea Berthelot reaction [17] and Jaffe's technique [18], respectively. Sodium, potassium, and chloride levels were estimated using the ion selective electrolyte (ISE) analyzer (AU600 Beckman Coulter®).

### 2.7. Statistical Analysis

Data collected were coded, entered into a computer, and cleaned. The statistical package GraphPad Prism-6 was used to analyze the data. Results were presented in tables using means and percentages. Chi-square, *P* value, and one-way ANOVA were used to assess the statistical significance. Statistical significance was decided when *P* < 0.05.

## 3. Results

Table 1 shows sociodemographic, obstetric, and biochemical characteristics of study participants. Gestational age (*P* = 0.002), urea (*P* = 0.0003), and creatinine (*P* < 0.0001) were associated with malaria infection. However, age, gravidity, parity, sodium, and potassium showed no significant association with malaria infection.

Table 2 shows biochemical parameters in relation to degree of parasitaemia and no malaria. There were significantly increased plasma urea and creatinine with respect to increasing degree of parasitaemia (moderate and high) compared to nonmalaria group (*P* < 0.05 and *P* < 0.0001, resp.). However, the electrolytes showed no significant differences.

There was a significant difference in levels of urea and creatinine and the degree of parasitaemia when stratified according to the gestational age (Table 3). However, parity and gravidity showed no significance difference.

## 4. Discussion

This study investigated the effect of malaria infection on renal function in pregnant women in New Juaben Municipality, a mesoendemic area of seasonal malaria transmission [19]. Our findings showed an association between malaria and levels of urea and creatinine in pregnancy. This was more prominent among malaria infected pregnant women in their third trimester.

In contrast to other studies [10, 19, 20], this study showed no association with age, parity, and gravidity among those with malaria and the control group. However, a similar study [21] conducted in Sudan also revealed the same trend as observed in our study. Most of the cases were in their second and third trimesters (72%) while majority of the controls were in their first and second trimesters (90%). The increasing degree of parasitaemia with increasing gestational age reported in this study may be due to the immunocompromised state and the placental development during pregnancy thus allowing parasitic infection to occur [22].

Reports on the levels of urea and creatinine among malaria patients are varied. Whereas others [21] reveal decreasing levels of these parameters among malaria patients, other studies [23], including our study, report elevated levels of urea and creatinine alongside increasing levels of parasitaemia. Elevated plasma urea and creatinine levels in malarious subjects suggest possible renal impairment [24] and this is further supported by the strong correlation of these parameters to the increasing degree of malaria parasitaemia. Meanwhile, the association of* Plasmodium falciparum *infection with clinically significant renal and renal related biochemical disorders has been previously established [25]. These disorders are thought to be mediated by a complex interaction of mechanical, immunologic, cytokine, humoral, and acute phase response and nonspecific factors and hemodynamic factors [26, 27].

Usually in normal pregnancy, there is a fall in plasma urea and creatinine concentration due to several factors, including the dilutional effect of an expanding plasma volume, decreased production (positive nitrogen balance), and increased renal excretion as a consequence of a pregnancy-induced increase in the glomerular filtration rate [28]. Despite all these considerations, serum urea levels do not reflect the performance of the kidneys like creatinine. This is because urea production is also affected by dehydration, food intake, and tissue catabolism. Thus an increase in serum urea concentration with a concomitant increase in serum creatinine concentration in the infected subjects as shown in this study suggests that the normal functioning of the kidneys has been compromised.

There was no significant difference (*P* > 0.05) in the levels of serum electrolytes of the malarious subjects compared with the control subjects. The study also showed no significant correlation between the intensity of infection and the electrolyte levels. This finding contradicts the findings of [21, 29] who reported electrolyte derangements among pregnant women, probably due to the variation in the pattern of malaria. However, Barber et al. [30] also found normal electrolyte levels among malarious pregnant women.

## 5. Conclusion

We conclude that malaria has a significant impact on renal biochemical profile (most importantly, urea and creatinine), with cases of increasing gestational age having a higher risk of the consequences of malaria. We recommend that pregnant women who report to hospitals with malaria should also be examined for kidney function. We further recommend that a larger sample size should be considered in future studies.

## Figures and Tables

**Table 1:** Demographic and biochemical characteristics of study participants.

Variable	Total *N* (%)	Malaria positive *N* (%)	Malaria negative *N* (%)	*P* value
Age (years)				0.0609
15–24	38 (38)	22 (44)	16 (32)	
25–34	38 (38)	15 (30)	23 (46)	
35–44	24 (24)	13 (26)	11 (22)	
Parity				0.3785
Nulliparous	40 (40)	18 (36)	22 (44)	
Primiparous	28 (28)	16 (32)	12 (24)	
Multiparous	32 (32)	16 (32)	16 (32)	
Gravidity				0.8501
Primigravida	27 (27)	14 (28)	13 (26)	
Secundigravida	29 (29)	15 (30)	14 (28)	
Multigravida	44 (44)	21 (42)	23 (46)	
Gestational age (Trimester)				^**∗**^0.0002
1st trimester	36 (36)	14 (28)	22 (44)	
2nd trimester	42 (42)	19 (38)	23 (46)	
3rd trimester	22 (22)	17 (34)	5 (10)	
Parasitaemia				N/A
low (<1000)	11 (22)	11 (22)	0 (0.0)	
medium (1000–9999)	28 (56)	28 (56)	0 (0.0)	
high (≥10,000)	11 (22)	11 (22)	0 (0.0)	
Urea (mg/dL)	17.51 ± 9.58	20.92 ± 11.9	14.1 ± 4.4	^*∗*^0.0003
Creatinine (mg/dL)	0.82 ± 0.26	0.92 ± 0.3	0.7 ± 0.1	^*∗*^< **0.0001**
Sodium (mmol/L)	148.50 ± 11.29	149 ± 11.7	0.09717	0.6405
Potassium (mmol/L)	4.13 ± 0.69	4.1 ± 0.8	4.2 ± 0.6	0.8759
Chloride (mmol/L)	104.5 ± 3.5	104.5 ± 3.2	104.5 ± 3.8	0.9438

Value are presented as *n* (%); frequency (percentage); mean ± SD. ^*∗*^Statistically significant. Parasitaemia was classified according to [13].

**Table 2:** Biochemical parameters in relation to degree of parasitaemia and no malaria.

Parameters	No malaria (0)	Degree of parasitaemia (parasites/*μ*L of blood)		
		Low (<1000)	Moderate (1000–9999)	High (≥10,000)
Urea (mg/dL)	14.1 ± 4.4	15.3 ± 2.7	16.32 ± 5.4^*∗*^	38.25 ± 13.6^*∗∗*^
Creatinine (mg/dL)	0.71 ± 0.1	0.73 ± 0.1	0.80 ± 0.2^*∗*^	1.42 ± 0.2^*∗∗*^
Potassium (mmol/L)	4.14 ± 0.5	4.12 ± 0.7	4.2 ± 0.8	3.9 ± 1.0
Chloride (mmol/L)	104.5 ± 3.8	104.3 ± 3.2	104.8 ± 3.6	104.1 ± 2.1
Sodium (mmol/L)	148 ± 11.0	147.1 ± 10.2	150.1 ± 10.9	148.3 ± 15.2

^*∗*^Significant difference from no malaria at *P* < 0.05. ^*∗∗*^Significant difference from no malaria at *P* < 0.0001.

**Table 3:** Biochemical Parameters and degree of parasitaemia stratified by gestational age, parity, and gravidae.

Parameter	Urea	Creatinine	Sodium	Potassium	Chloride	Parasitaemia
Gestational age						
1st trimester	16.25 ± 4.60	0.75 ± 0.16	146.91 ± 10.45	4.08 ± 0.76	104.72 ± 3.67	2016.714 ± 1673.14
2nd trimester	18.94 ± 9.93	0.93 ± 0.33	149.03 ± 10.51	3.97 ± 0.78	103.65 ± 2.09	5869.11 ± 5622.81
3rd trimester	26.79 ± 15.91	1.05 ± 0.37	150.78 ± 13.99	4.31 ± 0.86	105.02 ± 3.79	7367.35 ± 6068.71
*P* value	**0.033**	**0.032**	0.415	0.823	0.415	**0.016**
Parity						
Nulliparous	23.78 ± 14.86	1.02 ± 0.38	150.22 ± 14.46	4.22 ± 0.799	104.68 ± 3.57	6471.22 ± 5029.26
Primiparous	21.41 ± 12.66	0.93 ± 0.32	150.23 ± 10.52	3.81 ± 0.71	103.81 ± 1.98	6268.06 ± 5878.35
Multiparous	17.22 ± 5.36	0.80 ± 0.20	146.51 ± 9.30	4.28 ± 0.85	105.01 ± 3.85	3013.81 ± 4850.09
*P* value	0.277	0.139	0.585	0.194	0.561	0.119
Gravidae						
Primigravidae	23.47 ± 14.78	0.98 ± 0.34	153.01 ± 10.32	4.28 ± 0.78	104.50 ± 3.33	635272 ± 4808.03
Secundigravidae	21.30 ± 12.65	1.01 ± 0.41	146.18 ± 10.49	4.01 ± 0.83	103.86 ± 1.73	5693.63 ± 6069.92
Multigravidae	18.95 ± 9.25	0.82 ± 0.20	148.48 ± 13.94	4.11 ± 0.81	104.99 ± 3.84	3988.91 ± 5098.58
*P*-Value	0.551	0.141	0.281	0.587	0.566	0.391
